# An Investigation of the Sodium Nitroprusside Effects on Serum Lipids in an Animal Model of Schizophrenia by the Magnetic Resonance Study

**DOI:** 10.1021/acsomega.4c07072

**Published:** 2024-11-25

**Authors:** João Guilherme de Moraes Pontes, João Victor Silva Nani, Banny Silva Barbosa Correia, Tássia Brena Barroso Carneiro Costa, Danijela Stanisic, Mirian A. F. Hayashi, Ljubica Tasic

**Affiliations:** † Laboratório de Química Biológica (LQB), Departamento de Química Orgânica, Instituto de Química, and INCT-Bio (CNPq), Universidade Estadual de Campinas (UNICAMP), Campinas, SP 13083-970, Brazil; ‡ Instituto Nacional de Ciência e Tecnologia Translacional em Medicina (INCT-TM, CNPq), Ribeirão Preto 14026, Brazil; § Departamento de Farmacologia, Escola Paulista de Medicina (EPM), Universidade Federal de São Paulo (UNIFESP), São Paulo, SP 04044-020, Brazil

## Abstract

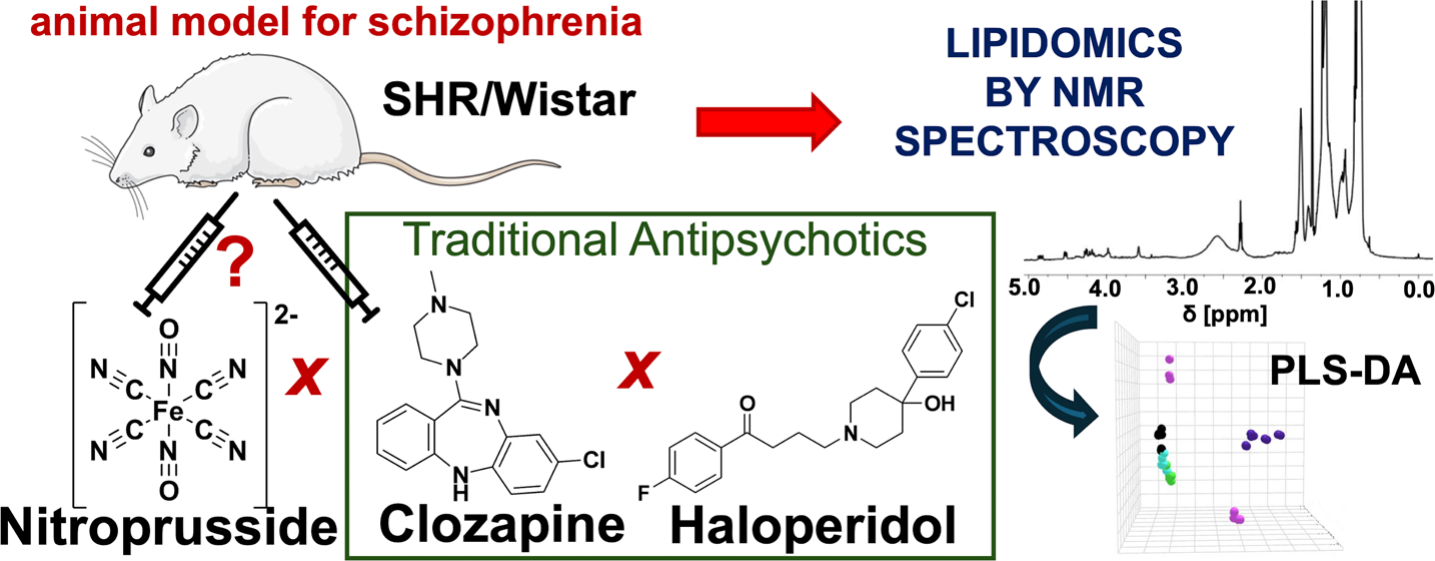

Schizophrenia (SCZ) is a multifactorial mental illness with limited knowledge concerning pathogenesis, contributing to the lack of effective therapies. More recently, the use of a nitric oxide donor named sodium nitroprusside (sNP) was suggested as a potential therapeutic drug for the treatment of SCZ. Despite the mixed results regarding the effectiveness of the sNP in reducing SCZ symptoms, successful trials on sNP in treatment-resistant SCZ were published. We have also demonstrated the power of evaluating the lipidic profiles of human clinical and animal model samples to identify the biomarkers of the pharmacological response to the diagnosis of mental disorders. Aim of this work is to evaluate the sNP effects in an animal model for SCZ studies through lipidomic profiles assessed by magnetic resonance spectroscopy (NMR). Lipidic profiling of serum from these animals indicated a more pronounced effect of sNP on lipids in the 0.50–6.00 ppm spectral region. Chemometric analysis also indicated an approximation of the lipidic profiling of SCZ animal model rats treated with sNP compared to that of the control group. In addition, we have compared the sNP treatment with other antipsychotics classically used in the clinic, such as haloperidol and clozapine, and the sNP treatment evaluated herein confirms the potential of sNP for the treatment of SCZ.

## 1 Introduction

The use of nitric oxide (NO) donor sodium nitroprusside (sNP) has been largely discussed in the literature as a potential therapeutic agent for the treatment of schizophrenia (SCZ).^1−4^ SCZ is a multifactorial illness, in which the blocking of glutamatergic *N*-methyl-d-aspartate (NMDA) receptors and consequent decrease in NO production may contribute to the pathogenesis of this psychiatric condition.^2,5^ NMDA receptors and NO also perform an important role in brain development and synaptic plasticity.^6^ The sNP can modulate the therapeutic target NMDA receptor with anxiolytic activity, and it was also identified as a promising adjunct treatment to reduce working memory impairment.^7^ Interestingly, NO released from sNP can cross the blood–brain barrier (BBB), and therefore, even peripheral intravenous infusion of sNP induces the release of dopamine in addition to activating NMDA receptors in the brain.^5,8,9^

While sNP has been used to treat acute hypertension since 1974,^10^ sNP is not a conventional medication for SCZ, and only more recently, it has been recognized as a promising alternative pharmacotherapy for treatment-resistant SCZ.^1,11,12^ Although still controversial,^13−16^ discrepancies in the reported experimental results could be due to the differences in the illness stage, disease duration, lifestyle, and age of the patients, among other factors.^8,12,17^ In addition, more recently, we have also demonstrated that the diagnosis biomarker for SCZ and other psychiatric conditions, named Ndel1 oligopeptidase, whose activity was demonstrated to be modulated by the pharmacological response to the treatment, was also modulated by the use of sNP as an adjunctive in the treatment of SCZ, with an interesting association with several aspects of clinical improvements in patients with SCZ.^12,18^

Different antipsychotics, such as clozapine,^19,20^ haloperidol,^19,21^ risperidone,^22,23^ and aripiprazole,^24,25^ among others, have also been used in animal models and also in patients to prevent or reverse SCZ-like behavior or symptoms, respectively. An animal model suggested as a reliable model for SCZ studies is spontaneously hypertensive rat (SHR) due to the presence of prominent features to study emotions and disturbances associated with SCZ, such as the deficit in contextual fear conditioning and duration of freezing responses against the aversive stimulus.^26^ In addition, SHR exhibits hyperlocomotion and reduced social behaviors, which could be reversed through the administration of antipsychotics.^27^ sNP was also tested in a dosage range varying between 0.3 and 6.0 mg kg^–1^ and evaluated SCZ-like animal behaviors.^18^ Herein, the effects of sNP were evaluated by a lipid profile study of serum samples from this SCZ animal model (SHR) and control normotensive Wistar rats (NWR) by nuclear magnetic resonance spectroscopy (NMR) analysis. Furthermore, the antipsychotic effects of sNP were also compared with those observed from clozapine and haloperidol treatment based on the lipid profiles. The present results bring new insights into the psychiatric field providing shreds of evidence pointing out the effective contribution of sNP in the treatment of SCZ.

## 2 Experimental Section

### 2.1 Animals

Male drug-naïve normotensive Wistar rats (NWRs) and spontaneously hypertensive rats (SHRs), aged between 4 and 5 months and weighing 250–300 g, were obtained from the in-house breeding colony at *Escola Paulista de Medicina* (EPM) from *Universidade Federal de São Paulo* (UNIFESP). The animals were accommodated in groups of four rats within each Plexiglas cage measuring 41 cm × 34 cm × 16.5 cm, which ensured a controlled environment with a temperature kept at 22–23 °C and a 12/12 h light/dark cycle (lights on at 07:00 AM), and with *ad libitum* access to standard rodent chow and water. All animal procedures adhered strictly to the guidelines outlined by the Committee on Care and Use of Laboratory Animal Resources (National Research Council, USA). Ethical approval for this study was obtained from the ethics committee of EPM/UNIFESP under CEUA certificate no. 7290170315.

### 2.2 Animal Treatment and Serum Collection

The administration of sNP to animals followed previously established protocols. Briefly, sNP (NITROPRUS—Cristália, SP, Brazil) diluted in 0.9% NaCl saline solution (vehicle) (1.0 mL kg^–1^) was injected by intraperitoneal (IP) route into adult (4 months old) NWR or SHR animals, with each group comprising of 4–6 animals, where SHR had been the animal model of schizophrenia. Blood samples were collected from the animals 4 h after the IP administration of either vehicle or sNP (2.5 or 5.0 mg kg^–1^). Clozapine and haloperidol were intraperitoneally administered in doses of 2.5 and 0.5 mg kg^–1^, respectively. Serum samples were obtained through blood centrifugation at 1000–2000*g* for 10 min at 4 °C. Subsequently, aliquots of serum were stored at −20 °C until further analysis, following previously described procedures.^19^

### 2.3 Sample Preparation and NMR Spectra Acquisition

NMR samples totalized 6 sample groups: 1) 4 samples from the SHR control group, 2) 5 samples from SHR + sNP (2.5 mg kg^–1^), 3) 4 samples of SHR + sNP (5.0 mg kg^–1^), 4) 4 samples from the Wistar control group, 5) 5 samples of Wistar + sNP (2.5 mg kg^–1^), and 6) 5 samples of Wistar + sNP (5.0 mg kg^–1^). Furthermore, for comparison of sNP with other antipsychotics (haloperidol and clozapine), 4 samples of Wistar + haloperidol, 5 samples of Wistar + clozapine, 5 samples of SHR + haloperidol, and 5 samples of SHR + clozapine were analyzed here.

The procedure for the extraction process and parameters used for NMR spectra acquisition and processing are according to the methodology previously reported.^19^ In detail, animal serum (0.5 mL) was mixed with 2.4 mL of the solvent mixture composed of methanol:chloroform:sodium chloride solution (0.15 mol L^–1^) in a ratio of 1:2:2 (v/v/v) for 1 min using a vortex. Then, the mixture was centrifuged for 20 min at 2200 *g*, at 10 °C, and the chloroform phase containing the serum lipids was carefully separated from the hydro-alcoholic phase. Chloroform was evaporated and stored at −20 °C until analysis by NMR.

Lipids (10 mg) were dissolved in 600 μL of 99.8% deuterated chloroform (CDCl_3_, Cambridge Isotope Laboratories Inc., Tewksbury, MA, USA), transferred into NMR tubes (5 mm), and kept at 4 °C to avoid chloroform evaporation and/or lipid oxidation.

All ^1^H NMR spectra were acquired in a Bruker Avance III NMR 600 MHz spectrometer equipped with a Triple Resonance BroadBand NMR probe (Bruker Corp., Billerica, MA, USA). ^1^H NMR spectra were recorded at 25 °C with an acquisition time of 1.32 s, a spectral window width of 12.335 Hz, a prescan delay of 12 μs, and 128 scans. Partial least-squares discriminant analysis (PLS-DA) was performed at the MetaboAnalyst platform^28^ using the spectral region between 0.50 and 6.00 ppm, excluding 1.50–1.68 ppm and 4.63–4.81 ppm for all spectra, to evaluate the effects of the different sNP dosages. For comparison of the sNP effects with those induced by clozapine or haloperidol, we performed chemometrics analysis using a spectral range between 1.30 and 2.60 ppm, excluding 1.50–1.68 ppm for all spectra. All analyses were done by using spectral bins with no processing mode. The NMR peak assignments were based on the literature.^19,29^

## 3 Results and Discussion

### 3.1 Evaluation of Sodium Nitroprusside (sNP) in Different Dosages

**Figure 1:**
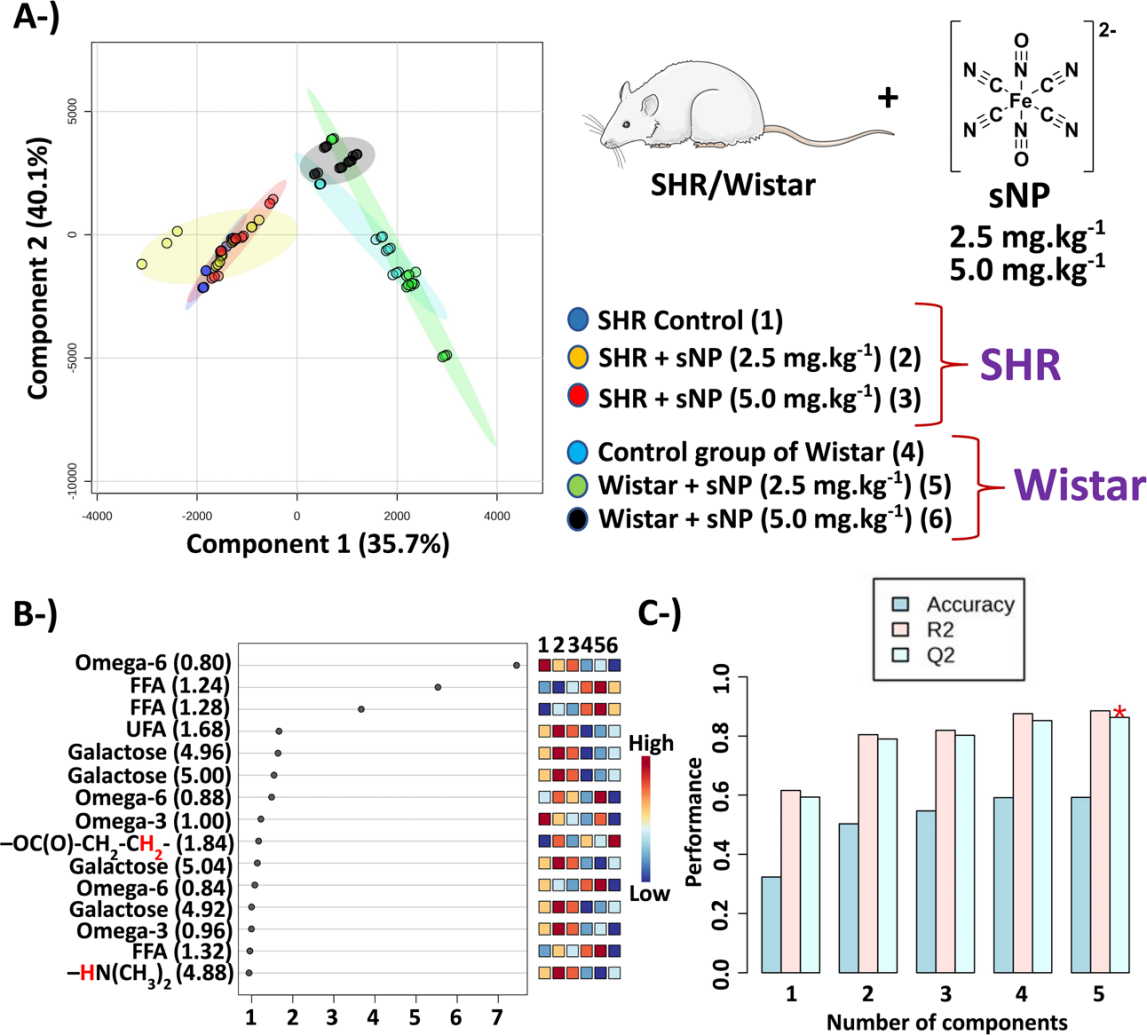
Results of the PLS-DA of the six groups. (A) Scores graph – 4 samples of the SHR untreated control group (dark blue color, group 1) + 5 samples of SHR + sNP 2.5 mg kg^–1^ (yellow color, group 2) + 4 samples of SHR + sNP 5.0 mg kg^–1^ (red color, group 3) + 4 samples of the Wistar untreated control group (cyan color, group 4) + 5 samples of Wistar + sNP 2.5 mg kg^–1^ (green color, group 5) + 5 samples of Wistar + sNP 5.0 mg kg^–1^ (black color, group 6) using a spectral range between 0.50 and 6.00 ppm with exclusion of 1.50–1.68 ppm and 4.63–4.81 ppm. (B) VIP scores. (C) The PLS-DA cross-validation with the accuracy of 59.3%; *Q*^2^ = 0.86 and *R*^2^ = 0.89 using 5 components. Abbreviations: FFA – free fatty acids; UFA – unsaturated fatty acids. P.S.: The image of the rat is available at the link smart.servier.com (free medical images).

Dosages of sNP of 2.5 mg kg^–1^ and 5.0 mg kg^–1^ were selected in this study to mimic conditions used with clinical inpatients reported in previous studies.^2^ The effects of sNP on lipid metabolism were evaluated by an NMR approach using different combinations of classes in chemometrics analysis – SHR control *vs* SHR + sNP (Figures 1 and S1–S4) and Wistar control *vs* Wistar + sNP (Figures 1 and S5–S8). The results of the PLS-DA indicated that a dose of 5.0 mg kg^–1^ of sNP caused more significant effects on lipids from serum than a lower (half) dose of sNP (2.5 mg kg^–1^) in the SHR (Figures 1, S4 and S9), while the effects in normotensive Wistar rats (NWR) were more pronounced for the lower dose of sNP (Figures 1, S8–S10). The higher dose of sNP needed to observe the effects in SHR may be related to the reported higher levels of blood nitric oxide of SHR.^30^

Results related to the SHR and Wistar (NWR) presented in the VIP scores of 6 groups (Figure 1c), and in the VIP scores shown Figures S1–S10, show that these chemometrics analyses corroborate and complement the discussion done herein. Chemometrics analyses were also performed using the total spectral region and other spectral ranges; however, δ of 0.50–6.00 was found more suitable because of the model fitting (*Q*^2^) and predictability (*R*^2^) values.

### 3.2 Normotensive Wistar Rats

Metabolites that indicated higher levels in NWR in comparison with SHR were FFAs and omega-6 fatty acids, where the highest FFA levels occurred in Wistar + sNP (2.5 mg kg^–1^) and the Wistar control, respectively. Results that indicated significant statistical differentiation were polyunsaturated fatty acids (PUFAs, δ = 2.70–2.84), glycerol, unsaturated FA chains, choline glycerophospholipids (ChoGPL, δ = 3.60–3.80), cholesterol (δ = 0.58–0.70), among others (Figures S6 and S7).

In the NWR model, a closer approximation was observed between the lipid profiles of the sNP-treated animals and the control group when 2.5 mg kg^–1^ of sNP was administered, indicative of the dose-dependent adjustments of the FFA levels. Furthermore, reduced cholesterol levels were observed, which contrasts with other antipsychotics’ effects that tend to increase cholesterol levels and provoke weight gain in long-term use.^31^

Glycerophospholipids (ChoGPL) perform different biological functions for the development of neural membranes such as stability, fluidity, permeability, and vital biochemical processes.^32,33^ However, disturbance in glycerophospholipids pathways has been associated with a dysfunction of mental illnesses such as schizophrenia and bipolar disorder^34^ and with neurodegenerative diseases.^33^ In our analysis, ChoGPL was significantly increased in Wistar + sNP (5.0 mg kg^–1^), which was a less effective dose in the treatment of NWR animals.

### 3.3 Spontaneously Hypertensive Rats

According to the VIP scores (Figures 1 and S9), we observed increases in the levels of unsaturated fatty acids (UFAs, δ = 1.60–1.70), omega-3 fatty acids (δ = 0.93–1.02), free fatty acids (FFAs, δ = 1.20–1.40), galactose (δ = 4.90–5.00), and amine protons (−**H**N(CH_3_)_2_) (δ = 5.20–5.40) in SHR + sNP (2.5 mg kg^–1^) and SHR + sNP (5.0 mg kg^–1^), while omega-6 fatty acids (δ = 0.75–1.00) were higher in the SHR control. In this sense, results that indicated a significant statistical differentiation (*p*-value < 0.05) were fatty acids, amino compounds, unsaturated fatty acid chains (−C**H**_2_–CH=CH–, protons in the α position; δ = 1.95–2.10), galactose, and glycerol (δ = 5.25–5.50) (Figures S2 and S3). The NMR peak assignments are shown in Figure S12 and Table S1.

Omega-3 and -6 are essential fatty acids obtained in the diet. A decrease in omega-6 levels in SHR administered with sNP may be related to the different biochemical roles such as cellular mediators, biochemical signaling, and precursors in the biosynthesis of other fatty acids.^35^ It is reported in the literature that omega-3 and unsaturated fatty acids show cardioprotective properties and enhance vasodilation.^36,37^

Glycerol was significantly increased after the administration of sNP, which corroborates with results related to the administration of other antipsychotics that were previously reported.^19^ Through the not very well-understood role of glycerol during the sNP treatment, it is suggested that glycerol and FFAs are produced due to the lipolysis process.^38^

Elevated amino compound levels in SHR + sNP in the blood could be due to the release of catecholamines and neurotransmitters in *locus coeruleus*, which posteriorly would be transferred to blood circulation and contribute to increased blood pressure,^39^ once amino compounds were detected in lower concentrations in NWR (Figure 1c).

### 3.4 Effectiveness of sNP Compared with Clozapine and Haloperidol

Haloperidol is a typical antipsychotic drug also widely prescribed in many countries for patients with SCZ,^40^ delirium,^41^ Huntington’s disease,^42^ and other illnesses. Among the side effects associated with the use of haloperidol are extrapyramidal symptoms (EPS), sedation, orthostatic hypotension, and weight gain.^43^ Some studies reported clozapine as more effective drug than haloperidol in reducing hostility and aggressive behaviors for the treatment of psychoses^44,45^ and in controlling episodes of SCZ.^19^

Clozapine is an atypical antipsychotic drug used in different brain disorders and neurological diseases, including SCZ, major depressive disorder (MDD), and Parkinson’s disease.^46^ It was developed in the late 1950s and became known principally due to the production of minimal or total absence of EPS, which is associated with muscular and movement dysfunctions.^47^ In the 1980s, the efficacy of clozapine in patients with SCZ and with resistance to other treatments was reported, leading to its approval by the Food and Drug Administration (FDA, United States of America) in 1990 and worldwide dissemination.^46,48,49^ Although clozapine is licensed in many countries, there are a variety of risks and side effects associated with this antipsychotic, which has led to the establishment of different regulations by many countries.^49^ In this sense, agranulocytosis induced by clozapine is the most known risk reported in the literature, which also led to its withdrawal from the market in the 1970s in Finland.^46,50^ Furthermore, clozapine is associated with cardiotoxicity,^51,52^ seizures,^53^ pneumonia,^54^ obsessive-compulsive symptoms,^54,55^ and even suicide and an increased risk of death.^46,51,56^

Despite the side effects related to the administration of sNP, such as bradycardia, dizziness, and hypotension, among others, besides a couple of specific contraindications,^10^ previous studies have reported a mode of action faster for the sNP in treatment-resistant SCZ than other antipsychotics, mainly in younger patients.^1,11^ Titulaer et al.^11^ suggested the administration of sNP in low doses as an adjunct for therapy with other antipsychotics, since high dose and prolonged administration could cause proarrhythmia.^11,57^

**Figure 2:**
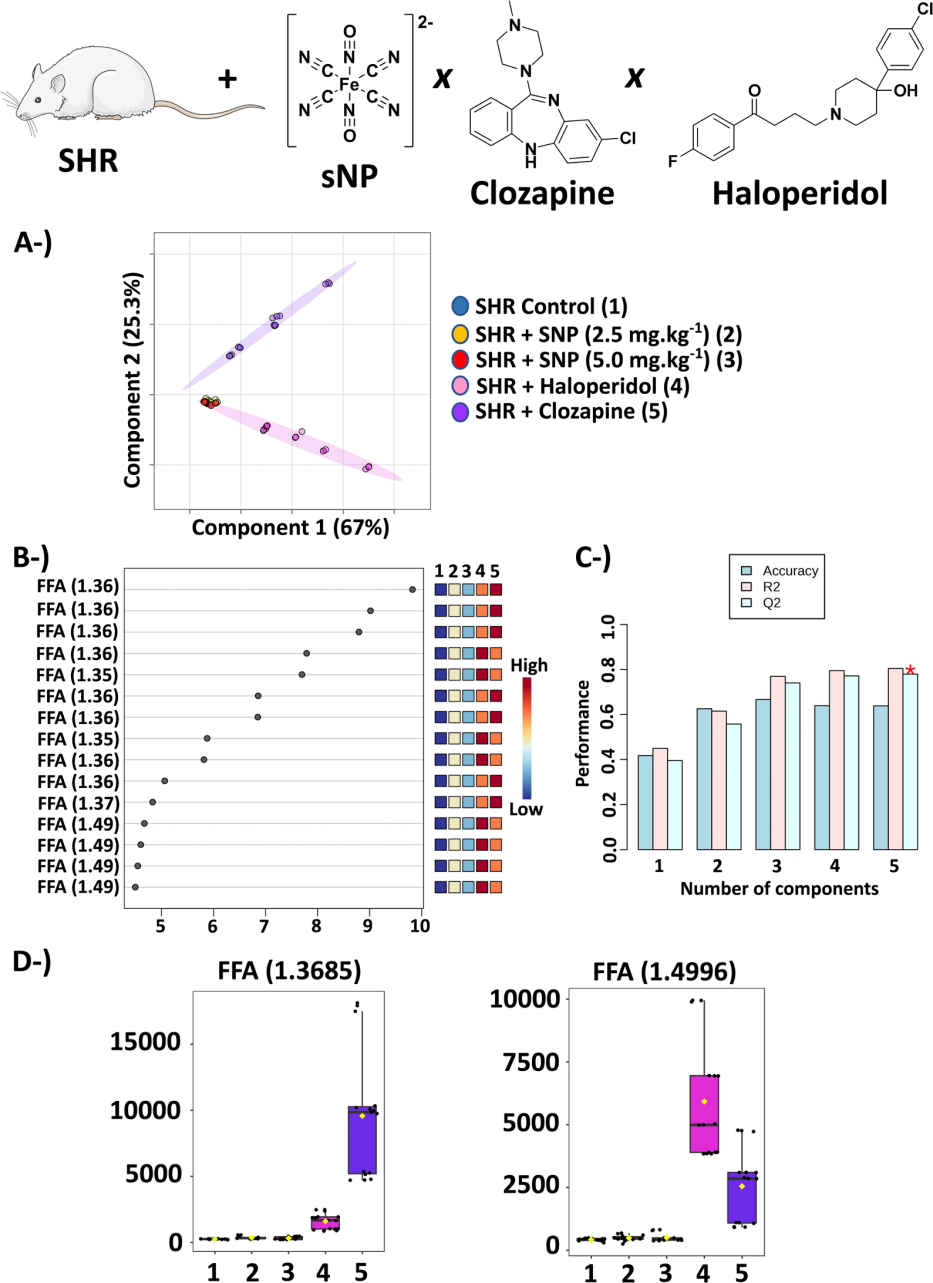
The results of the PLS-DA of SHR treated with sNP, haloperidol, and clozapine. (A) Scores graph (2D) – 4 samples of the untreated SHR control group (dark blue color, group 1) + 5 samples of SHR + sNP 2.5 mg kg^–1^ (yellow color, group 2) + 4 samples of SHR + sNP 5.0 mg kg^–1^ (red color, group 3) + 5 samples of SHR + haloperidol (pink color, group 4) + 5 samples of SHR + clozapine (purple color, group 5) using a spectral range between 1.30 and 2.60 ppm with exclusion of 1.50–1.68 ppm. (B) VIP scores. (C) The PLS-DA cross-validation with the accuracy of 63.8%; *Q*^2^ = 0.78 and *R*^2^ = 0.80 using 5 components. (D) Box plots of the original concentration of variables δ = 1.36 and 1.49, which were assigned to FFA. Abbreviation: FFA – free fatty acids.

Chemometric analysis of our NMR data indicated a higher similarity in the metabolic profiles of SHR (Figure 2) and NWR (Figure S11) control animals with those treated with sNP concerning those treated with clozapine or haloperidol.

Considerable increases in free fatty acid (FFA, δ = 1.20–1.40) levels were observed after the treatment with clozapine and haloperidol (Figures 2e and S11e, respectively) in comparison with animals treated with sNP. Alterations in fatty acid metabolism were reported by Canfrán-Duque et al.^58^ in an *in vitro* study of antipsychotics of the first and second generations.^58^ Furthermore, clinical studies have shown that treatment with certain antipsychotics (risperidone, olanzapine, and haloperidol, among others) favors PUFA (*n* – 3 and *n* – 6) biosynthesis through upregulation of related genes. So, these medications increase cardiac risks such as arrhythmias, since these PUFAs harm the signaling of different vital pathways—synaptic, immune, and inflammatory.^59^

Another study reported elevated serum FFA levels in patients with SCZ treated in the long term with chronic antipsychotics such as clozapine, which could be harmful, causing blood glucose metabolism disturbances and insulin resistance.^60^

### 3.5 sNP for the Treatment of SCZ Patients

Previously, the effects of typical and atypical antipsychotics such as haloperidol and clozapine were investigated to estimate biochemical responses as metabolic consequences of the administration of these drugs in animal models and also studied SCZ-like animal behaviors after sNP administration.^18,19^ Herein, we report for the first time alterations in lipid profiles after sNP administration and a comparative study of sNP with haloperidol and clozapine in the animal SCZ-model using lipidomics by NMR to evaluate how lipidic changes are reflected in the control animal and SHR (a reliable animal model for SCZ). These biochemical responses could provide insights into drug effects, which are necessary to a previous understanding of sNP administration in humans, since there is an issue about the resistance of patients to the use of traditional antipsychotics.^61,62^

An increase in FFA was observed after sNP administration (Figures 1, S1, and S2). However, this increase was even more pronounced when clozapine and haloperidol were administered (Figure 2). Cellular membranes of SCZ patients exhibit a deficit in phospholipids, which release FFA from the hydrolysis of these phospholipids.^63^ Therefore, the sNP treatment appears to be more suitable and less aggressive than other antipsychotics since it indicates a lower extent of cellular membrane damage. In a similar reasoning, dysregulation in PUFA levels could be related to the degradation of erythrocyte membranes, which has been reported in SCZ patients.^64^

Glycerol is another metabolite with increased levels after sNP (2.5 mg kg^–1^) treatment (Figure S2). In studies of schizophrenia and other diseases, the increased levels of glycerol are associated with lipolysis of triglycerides, which generate FFA and glycerol.^65,66^ In sNP (5.0 mg kg^–1^) treatment, this significant increase in glycerol levels was not observed, which indicates that sNP in a specific dosage could contribute to reducing triglyceride degradation.

Herein, we used a reliable animal model of schizophrenia (SHR animals)^26,27^ to study sodium nitroprusside effects as a potential antipsychotic drug in comparison to haloperidol and clozapine using an NMR-based lipidomics approach. In this sense, the spectral region (δ = 0.50–6.00) used in the PLS-DA led us to the assignment of NMR peaks to a set of metabolites, while VIP scores and box plots helped select which metabolites (FFA, PUFA, and glycerol) were important in this discrimination of groups.

## 4 Conclusions

Our studies using NMR-based lipidomics indicate a higher effectiveness of 5.0 mg kg^–1^ of sNP for the treatment of an animal model of SCZ compared with clozapine or haloperidol as presented in the results of chemometric analysis (Figures 1 and 2). The animal model for SCZ studies employed here was considered a reliable model for the psychiatric field of research due to the demonstrated predictive and constructed validity and with special strong predictive validity for pharmacological interventions. The spectral range that had higher contributions to the discrimination of different groups in PLS-DA was between δ of 0.50 and 6.00, which mainly reflects changes in FA, PUFAs, and glycerol as shown in VIP scores and box plots (Figures 1b, 2b,d, and S6). Therefore, based on the lipidic profiles observed in rats treated with sNP, we suggest the use of sNP for the treatment of patients with treatment-resistant SCZ, considering factors such as (adequate) dose and age of patients (20 to 30 years) and excluding contraindicated cases, is advantageous, since sNP proved to be more effective than clozapine or haloperidol, as evaluated by NMR-based lipidomics performed here.

## Supporting Information Available

PLS-DA of 2 groups using SHR as an animal model; PLS-DA of 2 groups using NWR as an animal model; PLS-DA of 4 groups using SHR and NWR; PLS-DA of Wistar treated with sNP, haloperidol, or clozapine; ^1^H NMR peak assignments (PDF)

## Abbreviations

NMDA: *N*-methyl-d-aspartate
NMR: nuclear magnetic resonance spectroscopy
NWR: normotensive Wistar rats
PLS-DA: partial least squares-discriminant analysis
SCZ: schizophrenia
SHR: spontaneously hypertensive rats
sNP: sodium nitroprusside
VIP: variable importance in projection
