# Supplementary material for: An Investigation of the Sodium Nitroprusside Effects on Serum Lipids in an Animal Model of Schizophrenia by the Magnetic Resonance Study
Source: ACS Omega. 2024 Nov 25;9(49):48480–7. doi: 10.1021/acsomega.4c07072 (PMC11635526; doi:10.1021/acsomega.4c07072)
Supplement: Supplementary file 1 — ao4c07072_si_001.pdf [file ao4c07072_si_001.pdf]

Supporting Information

An investigation of the sodium nitroprusside effects on serum lipids in an animal model for schizophrenia by magnetic resonance study

João Guilherme de Moraes Pontes[a], João Victor Silva Nani[b,c], Banny Silva Barbosa Correia[a], Tássia Brena Barroso Carneiro Costa[a], Danijela Stanisic[a], Mirian A. F. Hayashi[b,c*], Ljubica Tasic[a*]

[a]Laboratório de Química Biológica (LQB), Departamento de Química Orgânica, Instituto de Química, Universidade Estadual de Campinas (UNICAMP), Campinas, SP 13083-970, Brazil, and INCTBio
[b]Departamento de Farmacologia, Escola Paulista de Medicina (EPM), Universidade Federal de São Paulo (UNIFESP), São Paulo, SP 04044-020, Brazil
[c]Instituto Nacional de Ciência e Tecnologia Translacional em Medicina (INCT-TM, CNPq), Ribeirão Preto 14026, Brazil

*ljubica@unicamp.br
*mhayashi@unifesp.br

Summary

- PLS-DA using SHR as an animal model

→ 2 groups (control · SHR + sNP) (Figure S1) S3

→ Box plots (control · SHR + sNP 2.5 mg kg^{-1}) (Figure S2) S4

→ Box plots (control · SHR + sNP 2.5 mg kg^{-1}) (Figure S3) S5

→ 3 groups (control · SHR + sNP 2.5 mg kg^{-1} · SHR + sNP 5.0 mg kg^{-1}) (Fig. S4) S6

- PLS-DA using Wistar as an animal model

→ 2 groups (control · Wistar + sNP) (Figure S5) S7

→ Box plots (control · SHR + sNP 2.5 mg kg^{-1}) (Figure S6) S8

→ Box plots (control · SHR + sNP 2.5 mg kg^{-1}) (Figure S7) S9

→ 3 groups (control · Wistar + sNP 2.5 mg kg^{-1} · Wistar + sNP 5.0 mg kg^{-1})

(Fig. S8) S10

- PLS-DA using SHR and Wistar as animal models

→ 4 groups (SHR control · Wistar control · SHR + sNP 2.5 mg kg^{-1} S11

SHR + sNP 5.0 mg kg^{-1}) (Figure S9)

→ 4 groups (Wistar control · SHR control · Wistar + sNP 2.5 mg kg^{-1} · S12

Wistar + sNP 5.0 mg kg^{-1}) (Figure S10)

- PLS-DA (Wistar + sNP · clozapine · haloperidol) (Figure S11) S13

- Peak Assignments

→ SHR + sNP (δ 0.50 to 6.00) (Figure S12) S14

→ Table S1 S14

sNP 2.5 mg.kg^{-1}

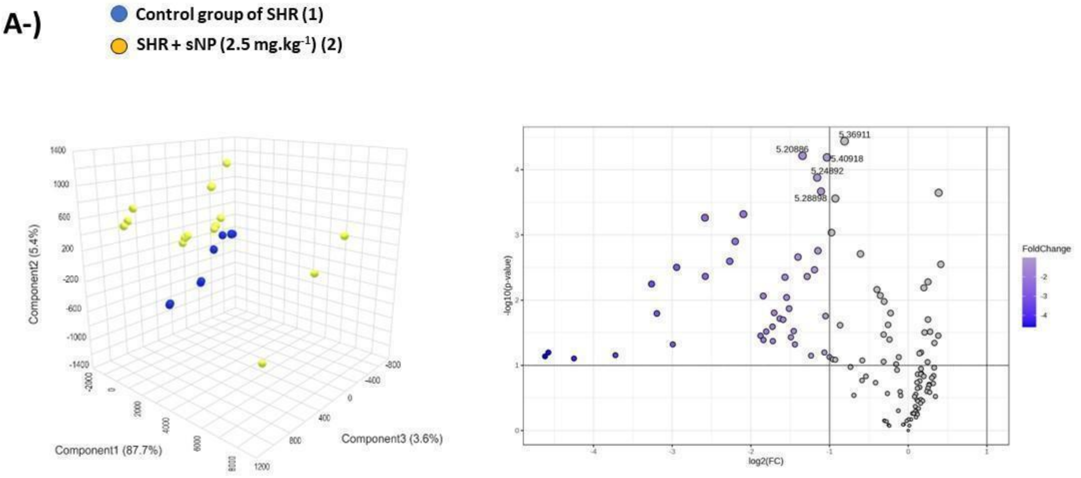

sNP 5.0 mg.kg^{-1}

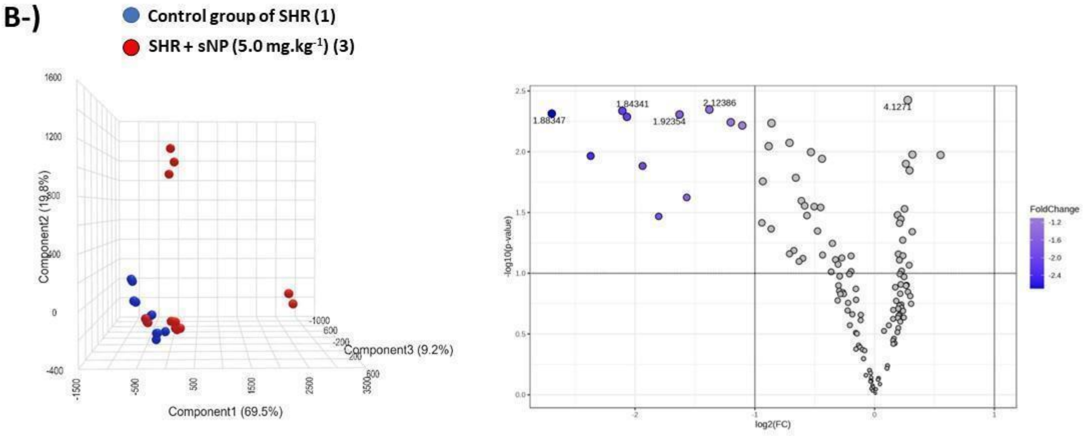

Figure S1. PLS-DA of two groups using a spectral range between δ 0.50-6.00 with the exclusion of δ 1.50-1.68 and δ 4.63-4.81. **A)** Scores graph with Volcano plot (40 results with statistical significance – *p*-value < 0.05) – 4 samples of untreated SHR control group (blue color, group 1) + 5 samples of SHR + sNP 2.5 mg kg^{-1} (yellow color, group 2). **B)** Scores graph with Volcano plot (11 results with statistical significance) – 4 samples of untreated SHR control group (blue color, group 1) + 5 samples of SHR + sNP 5.0 mg.kg^{-1} (red color, group 3).

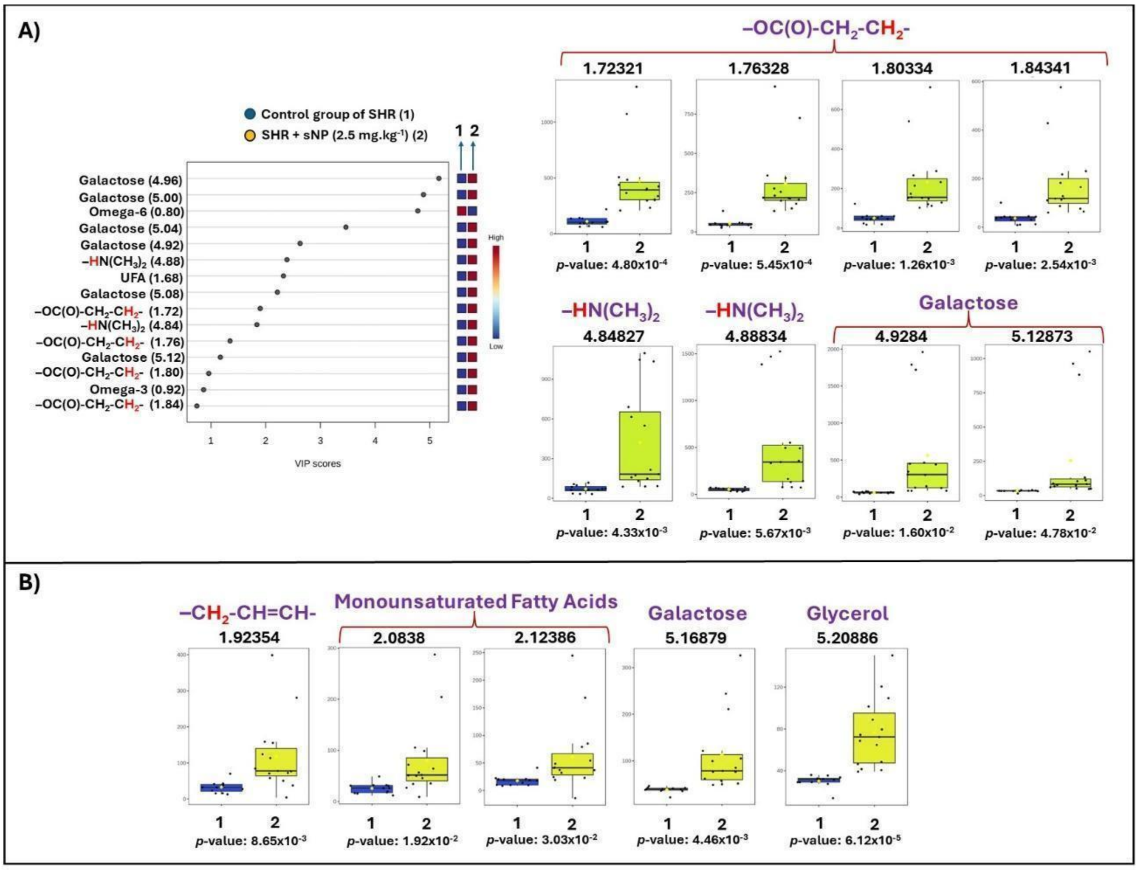

Figure S2. A) VIP scores graph of untreated SHR control group (blue color, group 1) · SHR + sNP 2.5 mg kg^{-1} (yellow color, group 2) with box plots with *p*-values < 0.05. **B)** Some other box plots with statistical significance on the spectral range between δ 0.50-6.00. Abbreviation: UFA – unsaturated fatty acids. The NMR peak assignments were done based on Correia *et al.* 2021[19] and Tynkkynen, 2012.[28]

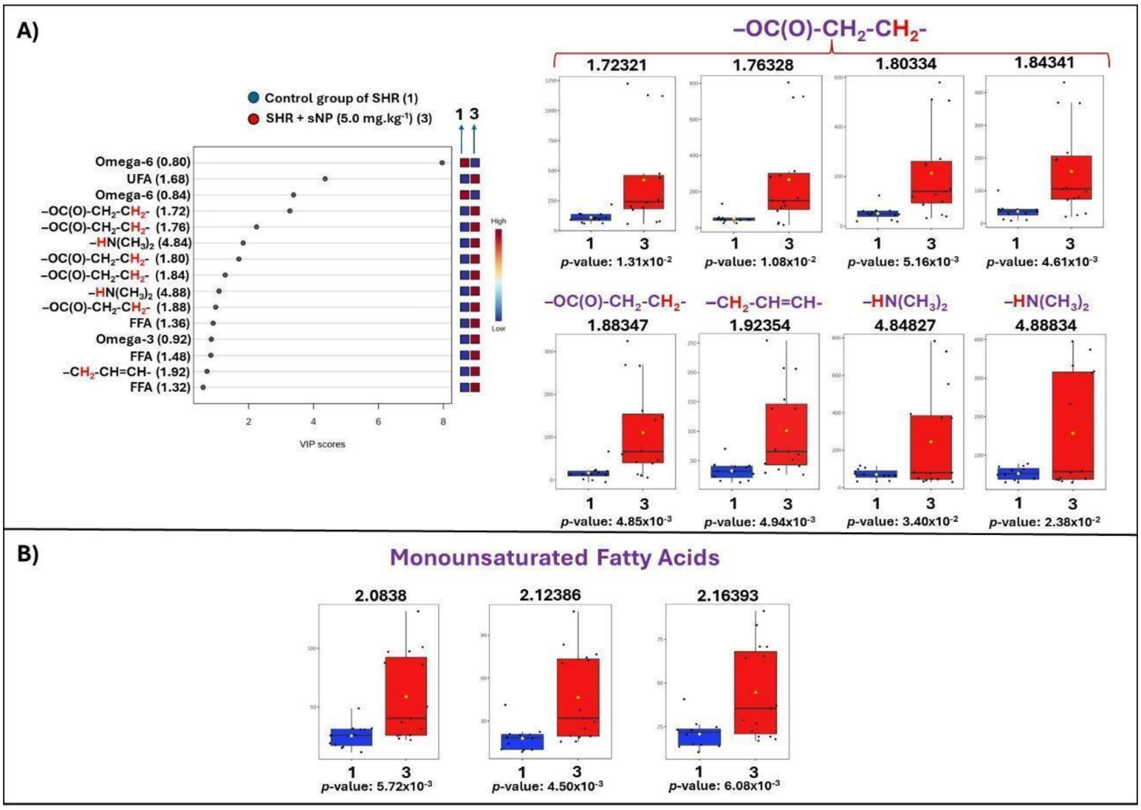

Figure S3. A) VIP scores graph of untreated SHR control group (blue color, group 1) · SHR + sNP 5.0 mg.kg^{-1} (red color, group 3) with box plots with p-values < 0.05. **B)** Some other box plots with statistical significance on the spectral range between δ 0.50-6.00. Abbreviations: FFA – free fatty acids; UFA – unsaturated fatty acids. The NMR peak assignments were done based on Correia *et al.* 2021[19] and Tynkkynen, 2012.[28]

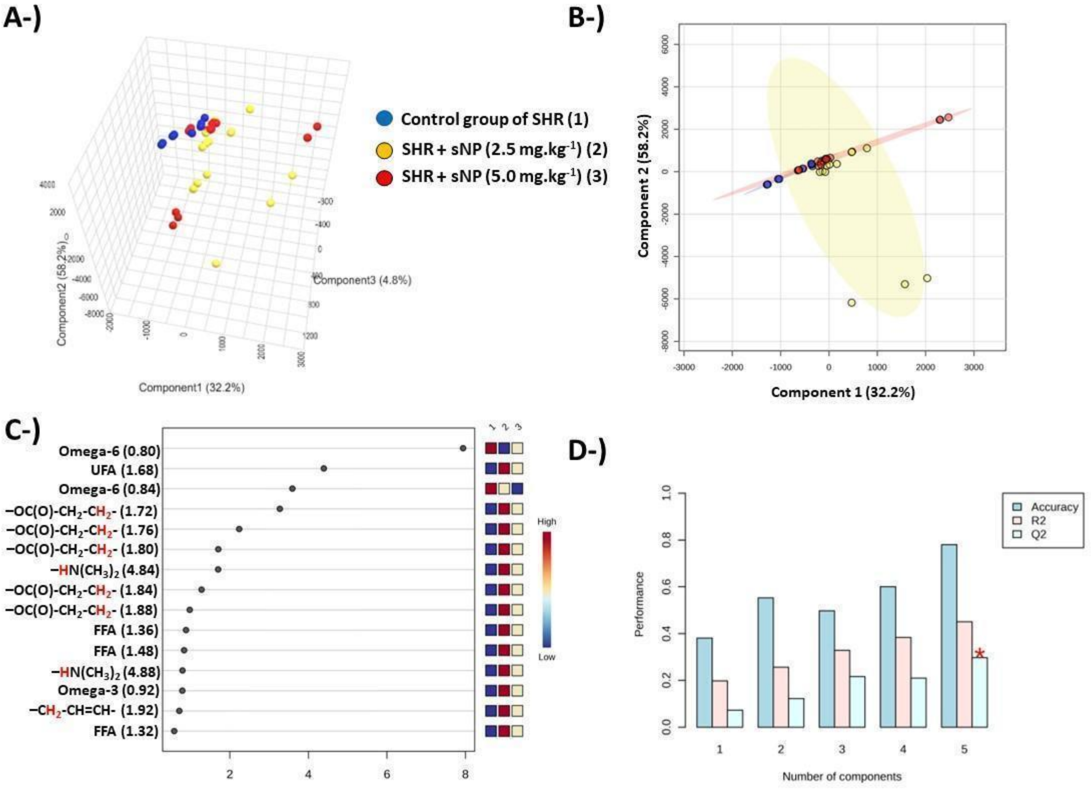

Figure S4. PLS-DA of three groups using the following spectral range δ 0.50-6.00 with the exclusion of δ 1.50-1.68 and δ 4.63-4.81. **A)** Scores graph – 4 samples of untreated SHR control group (blue color, group 1) + 5 samples of SHR + sNP 2.5 mg.kg^{-1} (yellow color, group 2) + 5 samples of SHR + sNP 5.0 mg.kg^{-1} (red color, group 3); Scores graph (2D) - PC1 (32.2%) · PC2 (58.2%); **C)** VIP scores. **D)** PLS-DA cross-validation with the accuracy of 78.0%; Q^2 = 0.29699 and R^2 = 0.45.072 using five (5) components. Abbreviations: FFA – free fatty acids; UFA – unsaturated fatty acids.

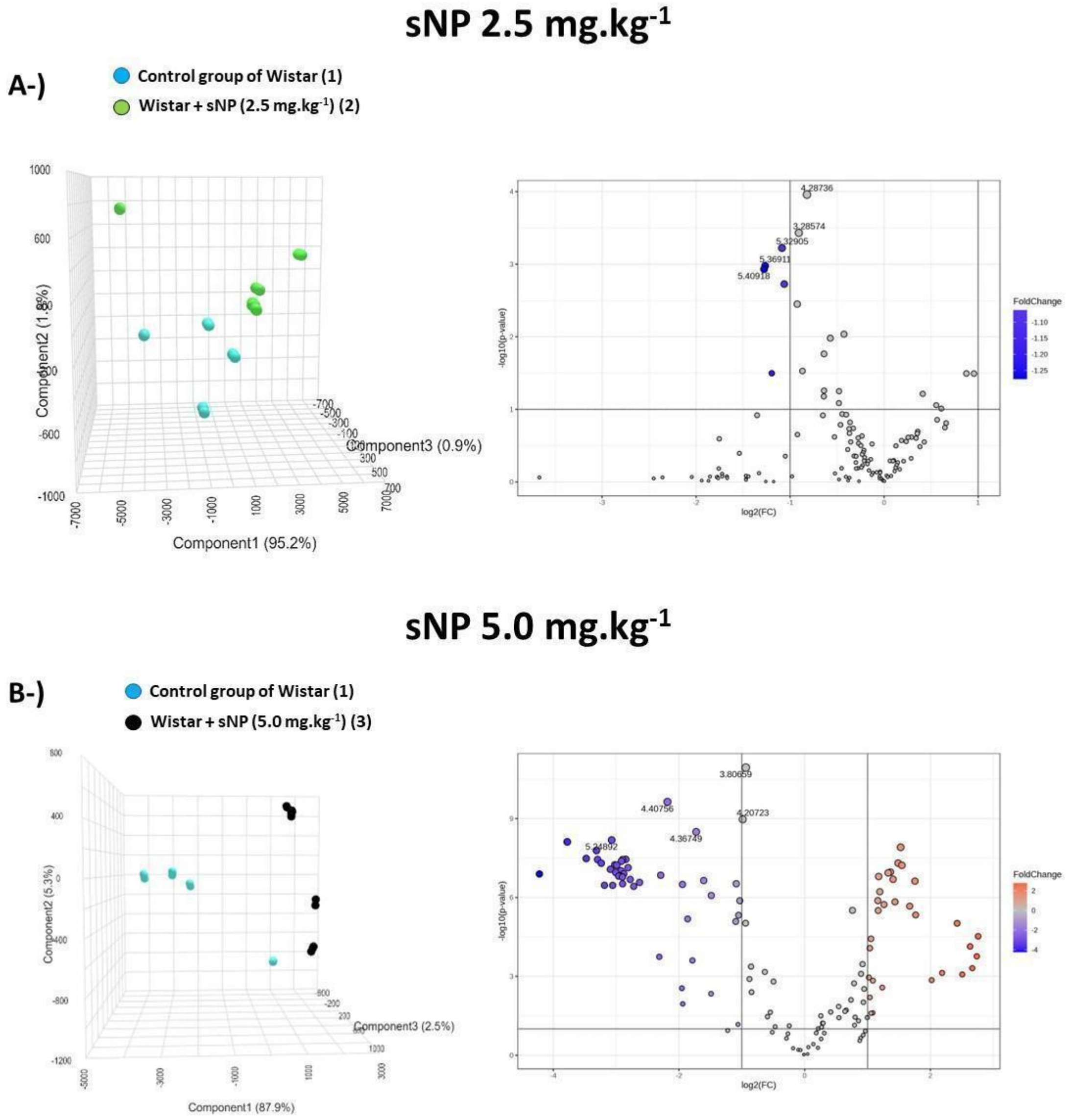

Figure S5. PLS-DA of two groups using a spectral range between δ 0.50-6.00 with the exclusion of δ 1.50-1.68 and δ 4.63-4.81. **A)** Scores graph with Volcano plot (5 results with statistical significance – *p*-value < 0.05) – 4 samples of untreated Wistar control group (cyan color, group 1) + 5 samples of Wistar + sNP 2.5 mg.kg^{-1} (green color, group 2). **B)** Scores graph with Volcano plot (74 results with statistical significance) – 4 samples of untreated Wistar control group (cyan color, group 1) + 5 samples of Wistar + sNP 5.0 mg.kg^{-1} (black color, group 3).

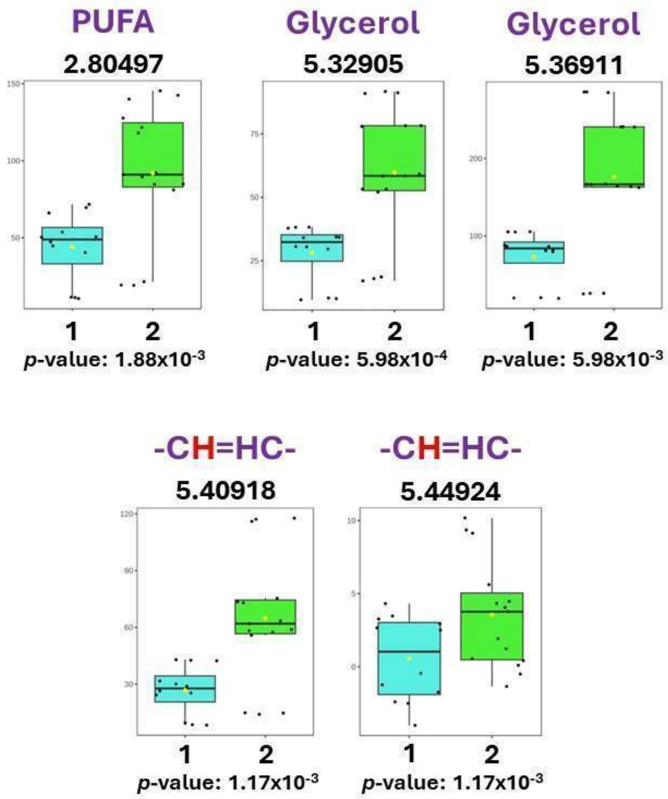

Figure S6. Box plots with statistical significance on the spectral range between δ 0.50-6.00 of untreated Wistar control group (cyan color, group 1) · Wistar + sNP 2.5 mg.kg^{-1} (green color, group 2). Abbreviation: PUFA – polyunsaturated fatty acids. NMR peak assignments were done based on Correia *et al.* 2021[19] and Tynkkynen, 2012.[28]

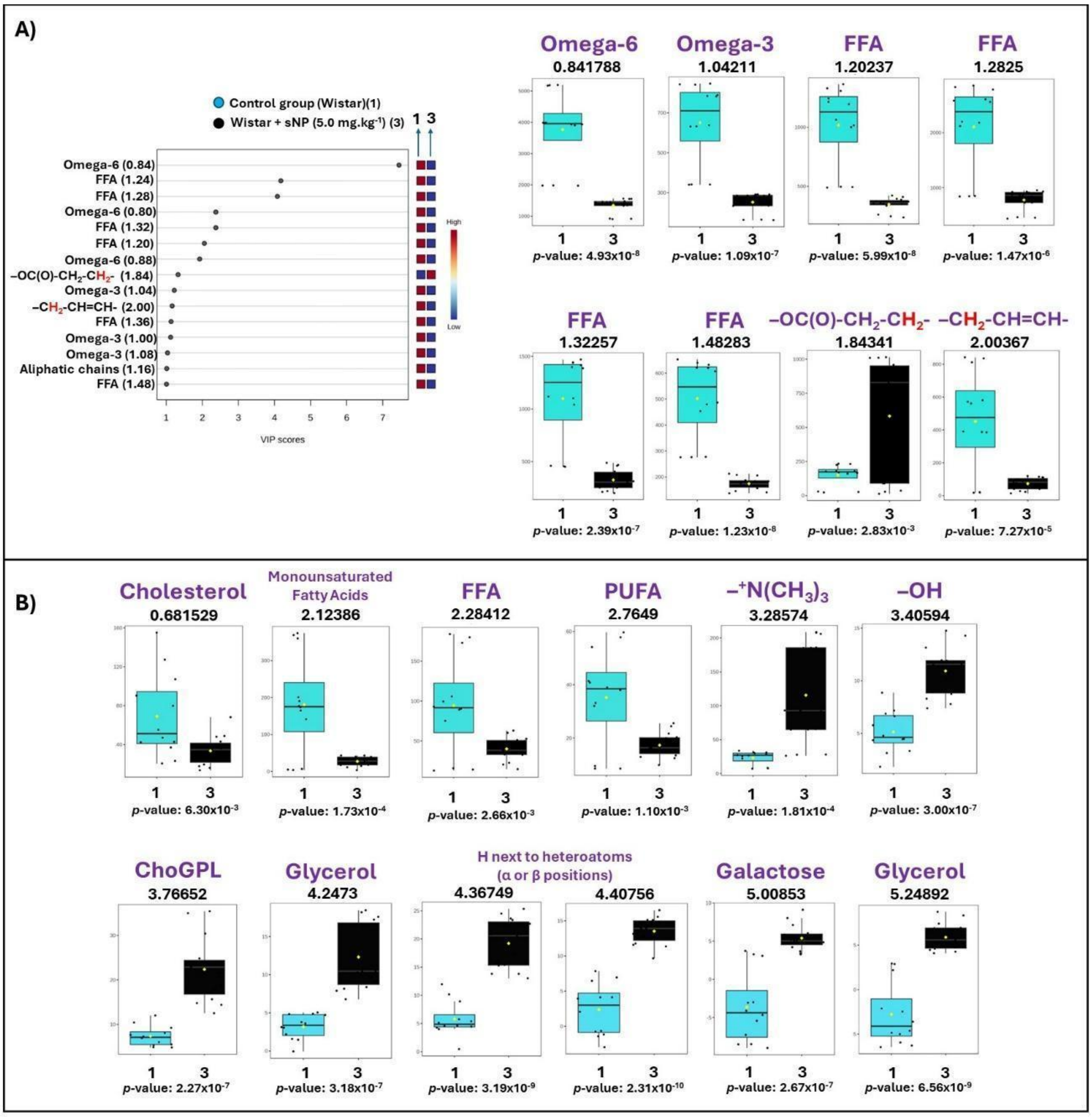

Figure S7. A) VIP scores graph of untreated Wistar control group (cyan color, group 1) · Wistar + sNP 5.0 mg.kg^{-1} (black color, group 3) with box plots with *p*-values < 0.05. **B)** Some other box plots with statistical significance on the spectral range between δ 0.50-6.00. Abbreviations: ChoGPL - choline glycerophospholipids; FFA – free fatty acids; PUFA – polyunsaturated fatty acids. The NMR peak assignments were done based on Correia *et al.* 2021[19] and Tynkkynen, 2012.[28]

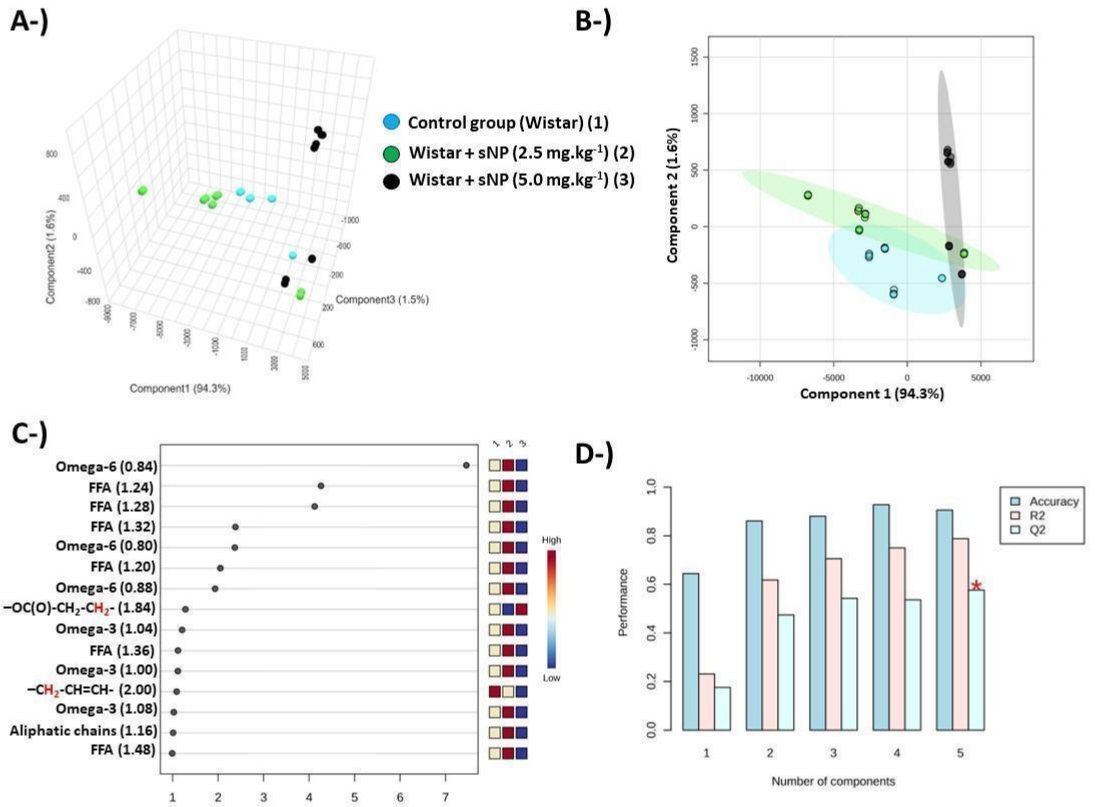

Figure S8. PLS-DA of three groups using the spectral range between δ 0.50-6.00 with the exclusion of δ 1.50-1.68 and δ 4.63-4.81. **A)** Scores graph (3D) – 4 samples of untreated Wistar control group (cyan color, group 1) + 5 samples of Wistar + sNP 2.5 mg.kg^{-1} (green color, group 2) + 5 samples of Wistar + sNP 5.0 mg.kg^{-1} (black color, group 3); **B)** Scores graph (2D) - PC1 (94.3%) · PC2 (1.6%); **C)** VIP scores. **D)** PLS-DA cross-validation with the accuracy of 90.5%; Q^2 = 0.57651 and R^2 = 0.7884 using 5 components. Abbreviation: FFA – free fatty acids.

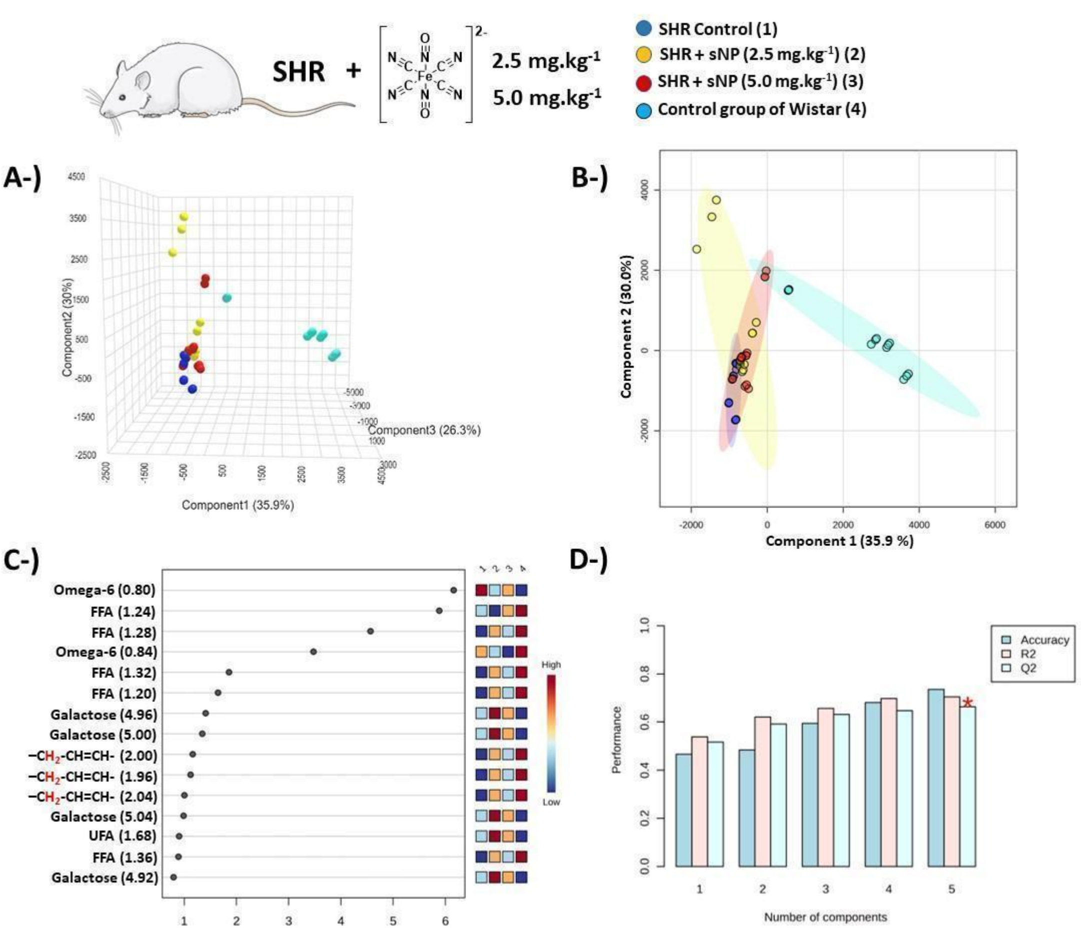

Figure S9. PLS-DA of four groups. **A)** Scores graph (3D) - 4 samples of SHR control untreated group (dark blue color, group 1) + 5 samples of SHR + sNP 2.5 mg.kg^{-1} (yellow color, group 2) + 5 samples of SHR + SNP 5.0 mg.kg^{-1} (red color, group 3) + 4 samples of Wistar control group (cyan color, group 4) using a spectral range between δ 0.50-6.00 with exclusion of δ 1.50-1.68 and δ 4.63-4.81; **B)** Scores graph (2D) - PC1 (35.9%) × PC2 (30.0%); **C)** VIP scores; **D)** PLS-DA cross-validation with the accuracy of 73.6%; Q^2 = 0.66345 and R^2 = 0.70458 using 5 components. PLS-DA results using Wistar are shown in Figure S5 in Supporting Information. Abbreviations: FFA – free fatty acids; UFA – unsaturated fatty acids.

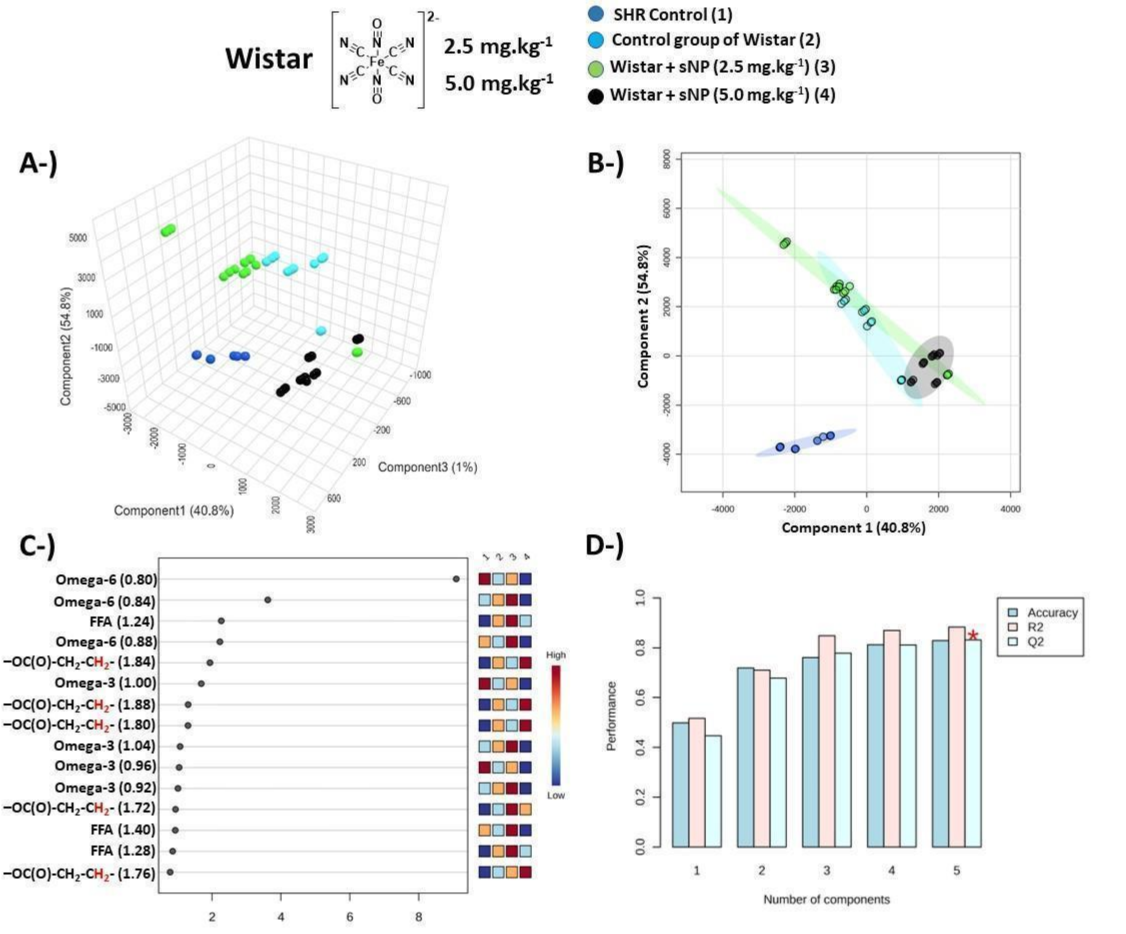

Figure S10. PLS-DA of four groups. **A)** Scores graph (3D) – 4 samples of untreated SHR control group (dark blue color, group 1) + 4 samples of untreated Wistar control group (cyan color, group 2) + 5 samples of Wistar + sNP 2.5 mg.kg^{-1} (green color, group 3) + 5 samples of Wistar + sNP 5.0 mg.kg^{-1} (black color, group 4) using a spectral range between δ 0.50-6.00 with exclusion of δ 1.50-1.68 and δ 4.63-4.81; **B)** Scores graph (2D) - PC1 (40.8%) · PC2 (54.8%); **C)** VIP scores; **D)** PLS-DA cross-validation with the accuracy of 82.9%; Q^2 = 0.83141 and R^2 = 0.88297 using five (5) component. Abbreviation: FFA – free fatty acids.

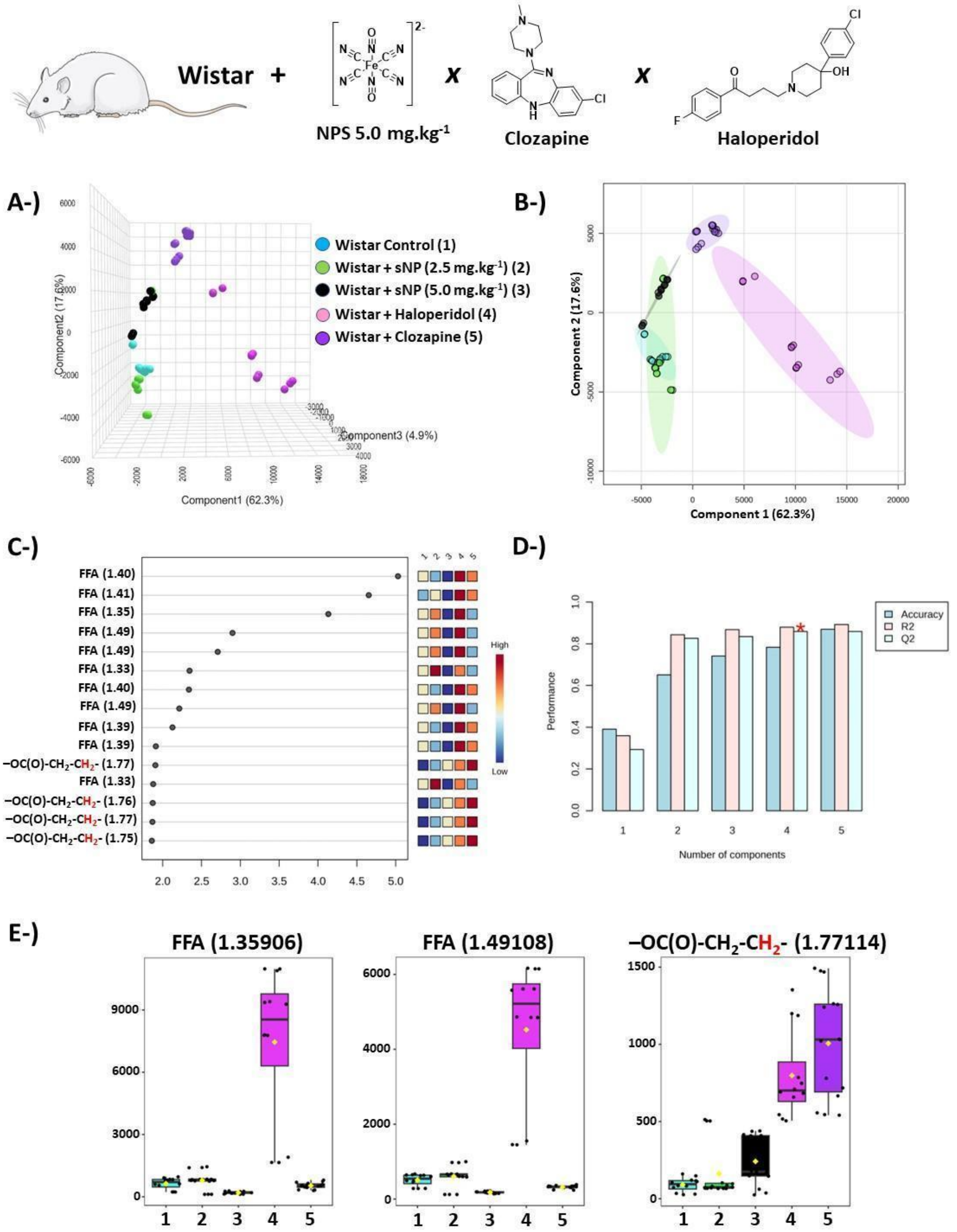

Figure S11. PLS-DA of Wistar treated with sNP, haloperidol, or clozapine. **A)** Scores graph (3D) - 4 samples of Wistar untreated control group (cyan color, group 1) + 5 samples of Wistar + sNP 2.5 mg.kg^{-1} (green color, group 2) + 5 samples of Wistar + sNP 5.0 mg.kg^{-1} (black color, group 3) + 4 samples of Wistar + haloperidol (pink color, group 4) + 5 samples of Wistar + clozapine (purple color, group 5) using a spectral range between δ 1.30-2.60 with exclusion of δ 1.50-1.68; **B)** Scores graph (2D) - PC1 (62.3%) · PC2 (17.6%); **C)** VIP scores; **D)** PLS-DA cross-validation with the accuracy of 78.3%; Q^2 = 0.8591 and R^2 = 0.8792 using 4 components. **E)** Box plots of the original concentration of variables 1.35 and 1.49, which were assigned to free fatty acids (FFA) and 1.77 assigned to β-methylene protons of the carbonyl (–OC(O)-CH_2-C**H₂**-). The NMR peak assignments were done based on Correia *et al.* 2021[19] and Tynkkynen, 2012.[28]

SHR + sNP

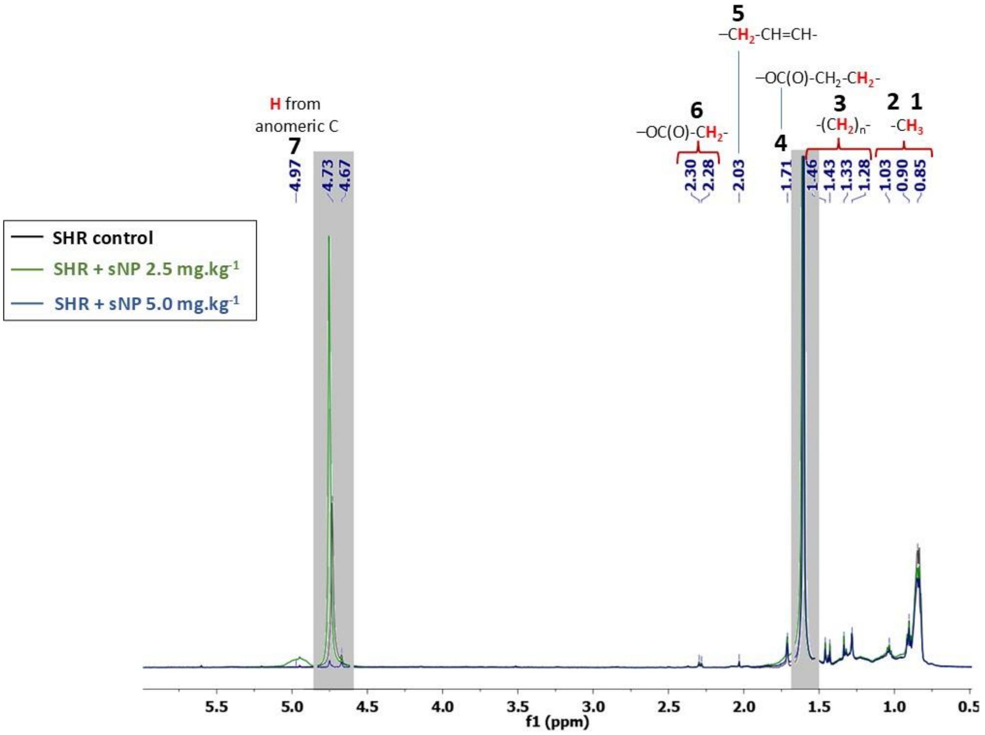

Figure S12. Metabolites assignment of ^{1}H-NMR spectra of untreated SHR control (black color), SHR + sNP 2.5 $mg.kg^{-1}$ (green color), and SHR + sNP 5.0 $mg.kg^{-1}$ (blue color) on the spectral range between δ 1.30 to 2.60. Spectral regions highlighted in gray represent the excluded area in chemometrics analysis – δ 1.50-1.68 and δ 4.63-4.81. The numbers from 1 to 7 indicate NMR peak assignments, which are related to protons in bold (Table S1) (Correia *et al.* 2021).[19]

Table S1. ^{1}H-NMR peak assignments (Correia *et al.* 2021)[19]

Peak	Chemical shift (ppm)	Assignment
1	0.75-1.00	-C**H₃** protons of saturated, oleic, and linoleic acyls (omega-6)
2	0.93-1.02	-C**H₃** protons of the linolenic chain (omega-3)
3	1.20-1.50	Methylene protons of aliphatic chains -(C**H₂**)$_n$
4	1.50-1.75	β-methylene protons of the carbonyl –OC(O)-CH_2-C**H₂**-
5	1.95-2.10	Methylene protons in the α position of double bonds –C**H₂**-CH=CH-
6	2.20-2.50	Methylene protons in the carbonyl α position –OC(O)-C**H₂**-
7	5.00	Anomeric carbon protons of galactose
